# Occurrence of putative *Culicoides* biting midge vectors (Diptera: Ceratopogonidae) inside and outside barns in Germany and factors influencing their activity

**DOI:** 10.1186/s13071-023-05920-z

**Published:** 2023-08-31

**Authors:** Sarah Groschupp, Helge Kampen, Doreen Werner

**Affiliations:** 1 Research Area 2 “Landscape Use and Governance”, Leibniz Centre for Agricultural Landscape Research (ZALF), Eberswalder Straße 84, 15374 Müncheberg, Germany; 2 Friedrich-Loeffler-Institut–Federal Research Institute for Animal Health, Südufer 10, 17493 Greifswald, Germany

**Keywords:** Arbovirus, Biting midges, Bluetongue virus, *Culicoides*, Obsoletus Group, Pulicaris Complex, Schmallenberg virus, UV-light trap, Vector

## Abstract

**Background:**

After several years without bluetongue disease, a ruminant illness caused by *Culicoides*-borne bluetongue virus (BTV), two new autochthonous cases were reported in 2018 in Germany. By contrast, Schmallenberg virus (SBV), another *Culicoides*-borne virus pathogenic to ruminants, has continuously circulated in Germany since its first emergence in 2011. The disease outbreaks have triggered numerous studies on the biology of the *Culicoides* vectors, but many ecological details are still obscure.

**Methods:**

*Culicoides* biting midge species were collected with UV-light traps on 10 farms in Germany, with one trap inside and one trap outside barns on each of the farms. Traps were run once a week for 24 h from January to December 2019. Collected biting midges were morphologically identified, counted and statistically evaluated, with a focus on the Obsoletus Group and the Pulicaris Complex of the ceratopogonid genus *Culicoides*, which are believed to contain the major virus vectors. Temperature and relative humidity recorded at each trap were linked to the quantity of caught *Culicoides*. Correlations between relative *Culicoides* abundance and presence of livestock or type of husbandry were also investigated.

**Results:**

A total of 38,886 *Culicoides* biting midges were trapped, with most of them belonging to the Obsoletus Group (51.0%) and the Pulicaris Complex (38.8%). The majority of captured specimens were collected in traps inside the barns. Obsoletus Group individuals were caught from late January to the last week of December while Pulicaris Complex individuals were captured from the end of March to early December. The lowest average temperatures at which members of the two groups were collected were 10.7 °C and 12.8 °C, respectively. While temperature had a statistically significant effect on the activity of both the Obsoletus Group and the Pulicaris Complex, relative humidity only significantly affected the activity of the latter. The presence of livestock significantly influenced the number of captured Obsoletus Group, but not of Pulicaris Complex specimens. Inside the barns, no statistical difference was found between numbers of caught Obsoletus Group and Pulicaris Complex specimens in livestock holdings with deep litter and manure scraper or slatted floor husbandry systems.

**Conclusions:**

The almost year-round presence of Obsoletus Group biting midges and the demonstrated high relative abundance of other potential* Culicoides* vector species inside barns suggest a high risk of indoor virus transmission to ruminants should BTV or SBV circulate locally. Appropriate structural, organisational and vector control measures to reduce biting midge exposure should be implemented.

**Graphical Abstract:**

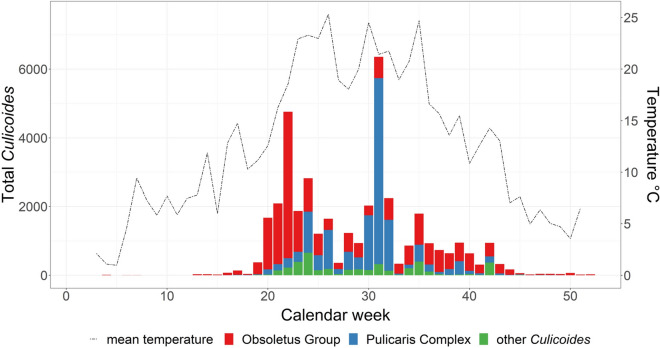

**Supplementary Information:**

The online version contains supplementary material available at 10.1186/s13071-023-05920-z.

## Background

Biting midges (family Ceratopogonidae) are distributed almost worldwide. Four genera of this family (*Culicoides*, *Leptoconops*, *Lasiohelea*, *Austroconops*) are known to contain species feeding on the blood of vertebrates [1]. With about 1300 described species, the genus *Culicoides* is the most important of these four genera from an infectiology point-of-view, comprising about 50 species that are potential vectors of disease agents. Major pathogens transmitted by members of the genus *Culicoides* include bluetongue virus (BTV; family Reoviridae, genus *Orbivirus*) and Schmallenberg virus (SBV; family Peribunyaviridae, genus *Orthobunyavirus*), both of which can cause severe infections in domestic and wild ruminants [1–4]. Bluetongue disease symptoms may include fever, lameness, muscle necrosis and facial oedema, and result in the death of the infected animals [5, 6], while Schmallenberg disease usually presents with milder symptoms such as fever, diarrhoea and a reduction in milk yield [7, 8]. In pregnant female ruminants, however, SBV infections often result in malformation of the offspring, stillbirths or abortions when occurring during a sensitive period of gestation [8, 9]. In addition to the negative effects on animal health, outbreaks of BTV will lead to restrictions on the trade of ruminants and their products. Vaccines are available for both BTV and SBV although they are administered restrictively unless in outbreak situations [10, 11]. Morbidity and mortality, trade restrictions and vaccination entail considerable costs and have a high economic impact on farmers and the livestock industry [8, 12, 13].

Until the outbreak in 2006 in central and northern Europe, BTV had circulated mainly in Africa, Asia and southern Europe where it had primarily been transmitted by *Culicoides imicola* Kieffer, 1913, an Afro-Asian species also established in the European Mediterranean area [14, 15]. The outbreak of bluetongue in central and northern Europe, where *C. imicola* was absent, suggested that native *Culicoides* species were competent to transmit BTV as well. Based on field and experimental findings, four members of the Obsoletus Group of the genus *Culicoides* (subgenus *Avaritia*: *Culicoides obsoletus* sensu stricto Meigen, 1818; *Culicoides scoticus* Downes & Kettle, 1952; *Culicoides dewulfi* Goethebuer, 1936; *Culicoides chiopterus* Meigen, 1830) and three members of the Pulicaris Complex of the same genus (subgenus *Culicoides*: *Culicoides pulicaris* sensu stricto Linnaeus, 1758; *Culicoides punctatus* Meigen, 1804; *Culicoides impunctatus* Goetghebuer 1920) were subsequently suggested as potential vectors [14, 16–19].

While no cases of BTV were recorded in Germany from 2010 to autumn 2018, two new cases were detected in cattle in southwestern Germany during routine BTV surveillance activities in December 2018 [13]. As a result, restrictions came into effect, and animals could only be moved from the affected region to unrestricted areas if they had been vaccinated against BTV or had a negative diagnostic test result (e.g. PCR, enzyme-linked immunosorbent assay [ELISA]) [13].

By contrast, SBV emerged in Germany and the Netherlands in 2011 as a completely novel virus spreading rapidly across central Europe [7, 20, 21]. It showed a phylogenetic similarity to viruses of the Simbu serogroup, which includes Akabane virus, a *Culicoides*-borne virus widely distributed in Asia and Africa [7, 22]. Due to the phylogenetic relatedness of the viruses, researchers concluded that *Culicoides* species were likely to transmit SBV as well [4, 7, 22–24]. Following the repeated detection of SBV in field-collected Obsoletus Group specimens in temperate European countries and laboratory evidence of virus susceptibility in both *C. obsoletus* and *C. scoticus* [25], some species of this biting midge group are now considered the main SBV vectors [22]. Unlike BTV, SBV has never disappeared from Germany and Europe but has been circulating cyclically since its first detection in 2011 [4, 9].

Despite the economic importance of *Culicoides* species as potential vectors of harmful pathogens, a research gap exists regarding distribution, biodiversity and vector competence of the species of this genus. Only a few studies have been conducted so far on the occurrence, phenology and composition of native *Culicoides* species in Germany and other European countries [e.g. 26–32]. The species mostly found in those studies were members of the Obsoletus Group and the Pulicaris Complex. The globally most important BTV vector, *C. imicola*, has so far only been detected in Europe in Mediterranean countries [17, 33] and once in southern Switzerland [34], but not further north.

Ultraviolet (UV)-light traps targeting adult biting midges are commonly used in studies on species distribution, activity and abundance of *Culicoides* [35, 36]. Biting midge monitoring usually focuses on livestock farms, where *Culicoides* species are most likely to occur, as they can find dung as a breeding substrate and vertebrates as hosts for a blood meal [37, 38]. In most of the studies examining *Culicoides* activity on farms, a higher number of *Culicoides* individuals were caught inside barns than outside these structures [26, 28, 33], whereas a greater species diversity was found outdoors [26, 30, 33, 39]. These observations could be attributed to temperature or biting behaviour (endo-/exophagy) of the species [40].

Knowledge about the occurrence, composition and ecology of biting midge vector species is still fragmentary. Efficient management of biting midges and biting midge-borne diseases, however, depends on detailed up-to-date data.

To contribute up-to-date data for Germany, the present study investigated the *Culicoides* species richness and relative abundance of the Obsoletus Group and Pulicaris Complex in relation to temperature, relative humidity and the presence of livestock inside and outside barns over the course of a calendar year on various German farms. Further, the influence of husbandry systems applied in barns on the occurrence of *Culicoides* was compared.

## Methods

### Collection sites

**Fig. 1:**
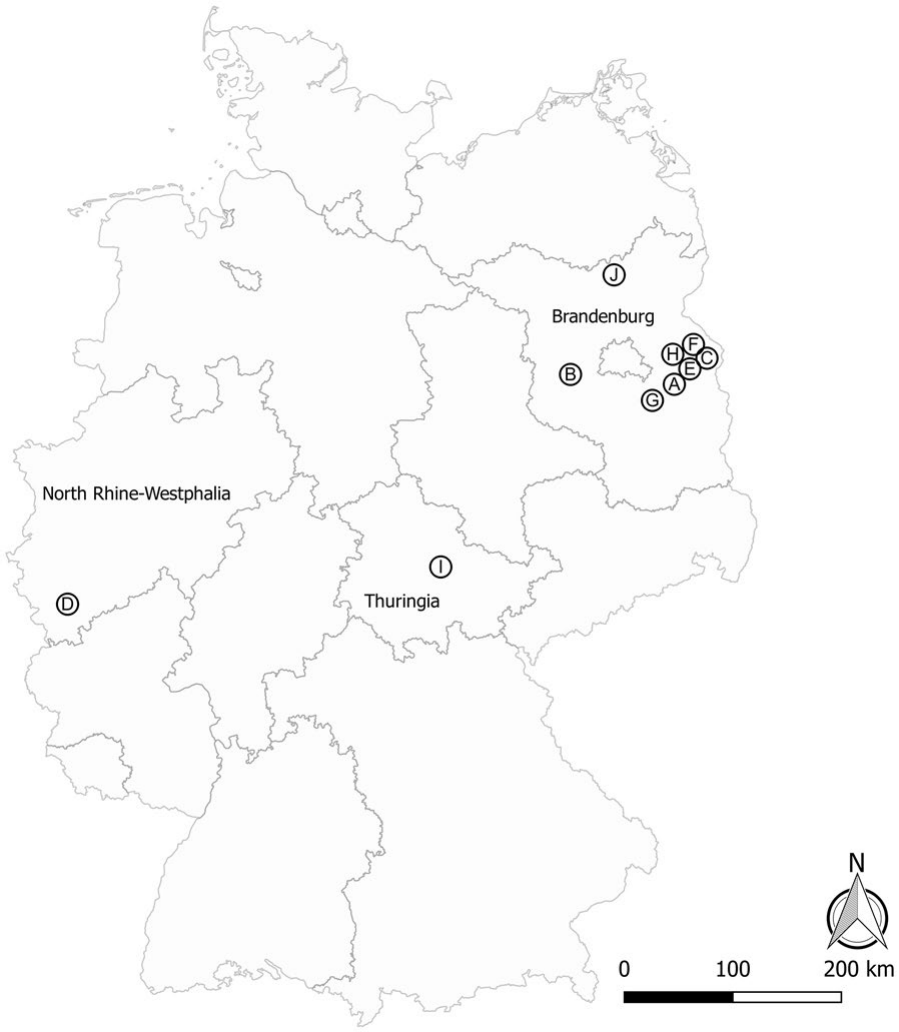
Map of Germany showing the geographical positions of the 10 study farms (A–J) in the federal states of Brandenburg, North Rhine-Westphalia and Thuringia. Letters correspond to the farm IDs listed in Table 1

Eight farms with ruminant livestock (cattle, sheep or goat) and two horse farms were selected as sampling sites for the collection of biting midges (Table 1). Of these 10 farms, eight were located in the German federal state of Brandenburg, and one farm each was situated in the states of Thuringia and North Rhine-Westphalia (Fig. 1). The selection criteria for the farms were: (i) at least 10 ruminants or horses kept on the farms; (ii) livestock kept indoors predominantly or all-year round; and (iii) distance between sampled farms at least 5 km. In addition, the selected farms should either have deep litter, be cleaned with a manure scraper or have integrated a slatted floor. The selected barns were solid buildings with large openings that allowed flying insects to enter and leave anytime.

**Table 1:** Characteristics and collection numbers for the 10 study farms

Farm ID	Study farm location	Average no. of farm animals	Livestock residence time in barns (months)	Husbandry system/frequency of barn-mucking	No. of collections (inside/outside)
A	Beerfelde	50 cattle	7	Deep litter/once a year	41/41
B	Groß Kreutz	400 cattle	12	Manure scraper/several times a day	49/48
C	Platkow	820 cattle	12	Manure scraper/several times a day	29/29
D	Zülpich	330 cattle	12	Slatted floor	45/45
E	Eggersdorf	15 sheep	12	Deep litter/twice a year	46/46
F	Quappendorf	60 sheep	10	Deep litter/once a year	44/45
G	Heidesee	10 horses	12	Deep litter/once a year	44/45
H	Müncheberg	150 horses	12	Deep litter/weekly	20/21
I	Eichelborn	40 goats	12	Deep litter/once a year	19/21
J	Lychen	150 goats	12	Deep litter/once a year	51/51
Total					388/392

### Trapping

BG-Sentinel UV-light traps (Biogents, Regensburg, Germany) were used to collect *Culicoides* biting midges once a week for 24 h from January to December 2019. Sampling was conducted when weather conditions were suitable for biting midge activity, i.e. no or only slight rain and wind. On each study farm, one trap was operated inside and one trap outside the barn, approximately 1.5–2 m (top edge of the trap) above the ground. Traps inside the barns were placed at a maximum distance of 5 m from livestock. The outdoor traps were usually located next to the doors or other openings of the barns but at a maximum distance of 50 m from the barns. Traps on the same farm were operated simultaneously. Caught insects were collected in beakers filled with 75% (v/v) ethanol and, after removal from the trap, stored in the alcohol in a dark environment until morphological identification.

### *Culicoides* identification

Collected biting midges were separated from other insects and identified under a stereomicroscope (Leica model M205 C; Leica Microsystems GmbH, Wetzlar, Germany) by wing patterns and other morphological characters, such as number of spermathecae, following the identification keys of Delécolle [41] and Mathieu et al. [42]. Because of the morphological similarity of some species (e.g. female *C. obsoletus* and *C. scoticus*), it was not possible to identify all individuals to the species level. If individual midges could not be identified to species, they were assigned to their species complex or group only.

All *Culicoides* were separated into females and males. Female *Culicoides* were further classified into engorged (hereafter referred to as ‘blood-fed’) and unengorged specimens.

### Climate data collection

Data loggers (EBI 20-TH1; Xylem Analytics, Ingolstadt, Germany) were attached to each trap to record temperature and relative humidity hourly throughout the entire study period. The recorded temperatures and relative humidities were averaged according to the calendar week. Threshold temperatures and relative humidity for the start and the end of seasonal biting midge activity were calculated from the weekly temperature of the first and last annual catches of the *Culicoides* species group/complex per trap.

### Influence of livestock and husbandry systems on the occurrence of *Culicoides*

To test whether the presence of livestock or the type of husbandry system in barns influenced the occurrence of *Culicoides*, the farmers provided information on livestock and farm management.

This study included seven deep litter barns, two with manure scrapers and one barn with a slatted floor (Table 1). In barns with deep litter, dung and urine fall onto a bed of straw. The straw is piled up repeatedly in the barn and accumulates over a long period (e.g. 1 year) [43]. In barns with manure scrapers, the area where the cattle defecates and urinates is cleaned several times a day by scraping the dung and urine into a pit [43]. In barns with slatted floors, the droppings disappear through slats into a pit below the slats [43]. In the latter two husbandry systems, only a bit of straw is present in the collection pits which are cleaned several times a year. Due to their similarities, these two husbandry systems were grouped together to analyse an impact on *Culicoides* activity, and compared with deep litter barns. Only barns mucked out once or twice a year were included in the analysis (Table 1). One of the seven deep litter barns was mucked out weekly, and excluded from this analysis.

### Data analyses

For data analysis, identified *Culicoides* individuals were grouped into Obsoletus Group (including *C. chiopterus*, *C. dewulfi*, *C. obsoletus* s.s., *C. scoticus*), Pulicaris Complex (including *C. pulicaris* s.s.,* C. punctatus*,* C. impunctatus*) and ‘other *Culicoides*’ (including all *Culicoides* species not belonging to the Obsoletus Group or the Pulicaris Complex).

Poisson generalised linear models (GLM) were used to check whether the numbers of caught Obsoletus Group and Pulicaris Complex specimens depended on temperature, relative humidity or the presence of livestock. The response variable was ‘biting midges counted’, explained by the predictors ‘temperature’, ‘relative humidity’ or ‘livestock presence’. The latter was a binary variable, set to ‘1’ when livestock was present and to ‘0’ when the livestock did not reside in the barns (c.f. Table 1).

To analyse the influence of the husbandry systems, the different barn sizes and the number of animals on the study farms had to be made comparable. To this end, the reference value of ‘livestock unit’ (LU), which facilitates the aggregation of animals of different species and ages and is based on the average annual number of animals on a farm [43], was employed to adjust livestock numbers. Also, the average number of *Culicoides* species group/complex per catch and farm was calculated for the indoor catches. These results were divided by the LU of each farm, thus guaranteeing meaningful and comparable statistics across farms. Two sample t-tests were used to compare the adjusted animal numbers between barns with deep litter and barns with manure scraper or slatted floor.

The data were managed in Microsoft Excel 2013 (Microsoft Corp., Redmond, WA, USA) and analysed and visualised in R version 4.1.3 [44] using the packages readxl [45], tidyverse [46] and ggplot2 [47], plm [48] and pglm [49]. In the calculation of whether the presence of livestock influenced the number of *Culicoides* caught, location-fixed effects were taken into account. For all calculations, *P*-values < 0.05 were considered significant.

## Results

A total of 38,886 biting midge specimens belonging to the genus *Culicoides* were captured on the 10 farms, of which 63.1% (*n* = 24,553) were trapped inside and 36.9% (*n* = 14,333) outside the barns. These data show that *Culicoides* were caught 1.7-fold more frequently inside the barns than outside.

**Fig. 2:**
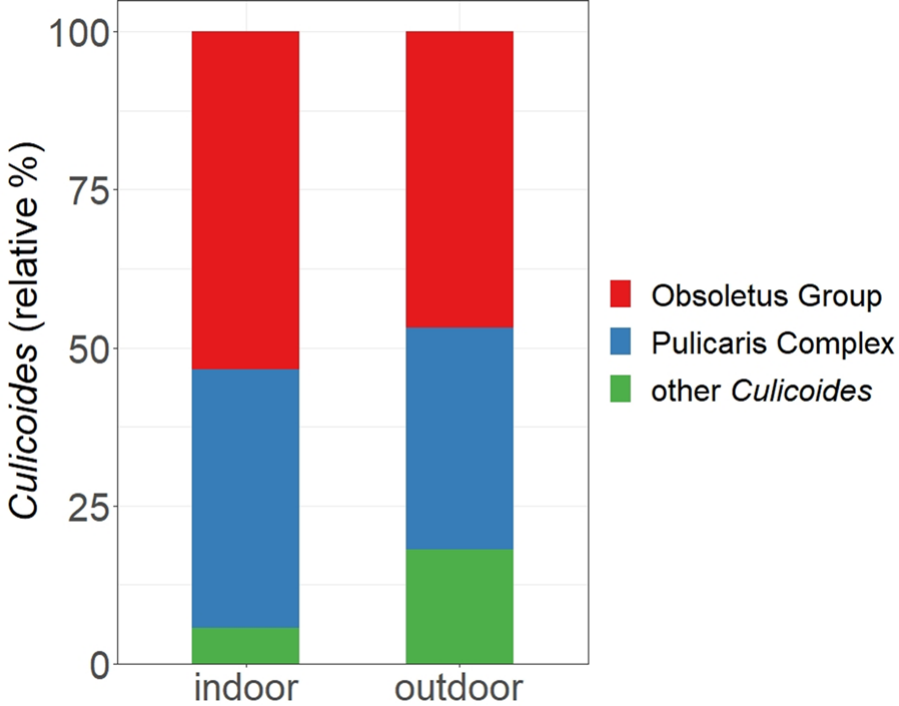
Relative percentages of indoor and outdoor catches of the Obsoletus Group, the Pulicaris Complex and ‘other *Culicoides*’

Overall, 24 different *Culicoides* species/species groups were determined morphologically, with 13 species/species groups trapped inside the barns and 23 species/species groups trapped outside the barns (Table 2). Of the captured *Culicoides* biting midges, 51.0% (*n* = 19,847) belonged to the Obsoletus Group, 38.8% (*n* = 15,072) belonged to the Pulicaris Complex and 10.2% (*n* = 3967) belonged to the classification ‘other *Culicoides’*. The relative proportion of biting midges per group/complex collected inside and outside the barns differed. More Obsoletus Group and Pulicaris Complex specimens were collected indoors than outdoors, while a larger proportion of ‘other *Culicoides’* was trapped outdoors than indoors (Fig. 2).

**Table 2:** List of *Culicoides* species collected inside and outside the barns

*Culicoides* species^a^	Inside barns^b^	Outside barns^b^
Obsoletus Group		
*C. chiopterus* ♂	−	+
*C. dewulfi* ♂	+	+
*C. obsoletus/C. scoticus*	+	+
*C. scoticus* ♂	+	−
Pulicaris Complex		
*C. impunctatus* ♀	−	+
*C. pulicaris*	+	+
*C. punctatus*	+	+
*Other Culicoides*		
*C. achrayi*	+	+
*C. albicans* ♀	+	+
*C. circumscriptus*	+	+
*C. duddingstoni* ♀	−	+
*C. fagineus*	+	+
*C. fascipennis* ♀	−	+
*C. festivipennis*	+	+
*C. griseidorsum* ♀	−	+
*C. kibunensis*	−	+
*C. nubeculosus*	+	+
*C. pallidicornis*	−	+
*C. pictipennis* ♀	−	+
*C. riethi*	+	+
*C. salinarius* ♀	−	+
*C. segnis*	−	+
*C. truncorum* ♀	−	+
*C. vexans* ♀	+	+

^a^Species within the groups are presented in alphabetical order. The morphologically determined specimens include both females and males, unless they are marked with ‘♀’ for females only or ‘♂’ for males only

^b^‘+’ indicates that a species was captured; ‘−’ indicates that the species was not captured

By far more females (94.2%, *n* = 18,689) of the Obsoletus Group were caught than males (5.8%, *n* = 1158); this also applied to the Pulicaris Complex (females: 99.1%, *n* = 14,932; males: 0.9%, *n* = 140) (Additional file 1: Table S1).

**Fig. 3:**
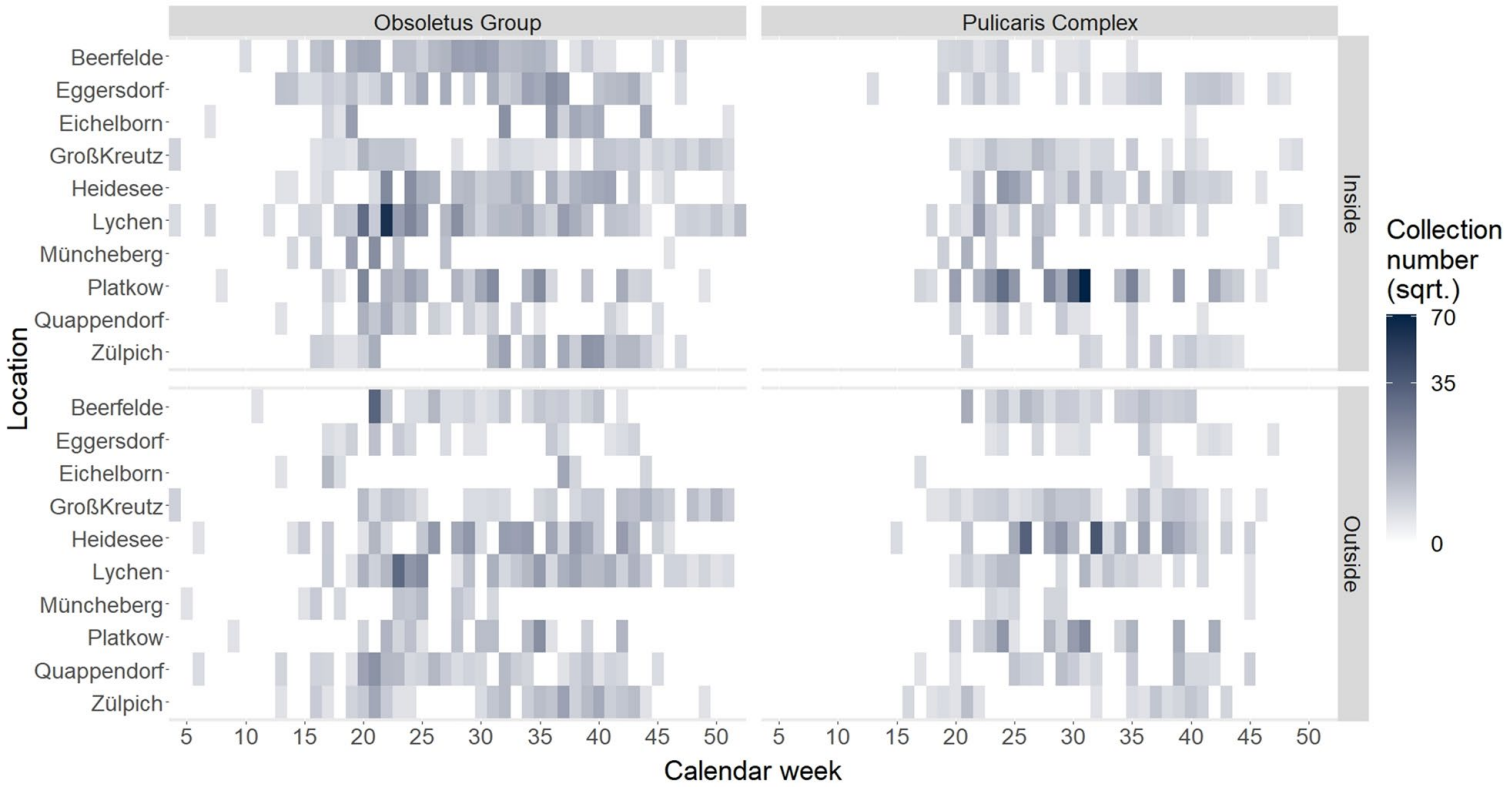
Heatmap showing the square root-transformed collection numbers of Obsoletus Group and Pulicaris Complex specimens for each farm per calendar week (columns). Position of the traps inside and outside of barns are displayed in the rows

Obsoletus Group biting midges were caught from calendar week 4 in January until calendar week 52 in December, both inside and outside the barns (Fig. 3). By contrast, Pulicaris Complex specimens were captured inside the barns from the end of March (calendar week 13) to the beginning of December (calendar week 49). Catches outside the barns containing Pulicaris Complex specimens occurred from calendar week 15 in the spring to calendar week 47 in the autumn. A heatmap (Fig. 3) shows an overview of the square root-transformed collection numbers of the Obsoletus Group and the Pulicaris Complex according to the location of the traps and the calendar week. A square root transformation presentation was applied due to the uneven distribution of the collection numbers and to show the differences between the catches from the different farms and traps more clearly [50].

The highest proportion (39.8%) of the total number of Obsoletus Group individuals was caught at the location Lychen (26.8% relative proportion) (Table 3). Of the Pulicaris Complex, 63.9% (69.1% relative proportion) of the individuals were collected in Platkow and 25.4% (20.3% relative proportion) in Heidesee (Table 3). At these two farms, the numbers of captured Pulicaris Complex individuals were higher than those of the Obsoletus Group. Particularly in calendar weeks 30 and 31 in Platkow and in calendar weeks 26 and 32 in Heidesee, the numbers of the Pulicaris Complex exceeded by far those of the Obsoletus Group (Fig. 3).

**Table 3:** Percentages and relative percentages of Obsoletus Group and Pulicaris Complex specimens caught at the respective study farms inside and outside the barns

Study farm	Obsoletus Group^a^			Pulicaris Complex^a^		
	Inside barns	Outside barns	Total	Inside barns	Outside barns	Total
Beerfelde	5.4 (4.6)	5.7 (7.1)	11.1 (11.7)	0.2 (0.2)	1.6 (1.8)	1.8 (2.0)
Eggersdorf	5.5 (4.8)	0.3 (0.5)	5.8 (5.3)	0.9 (0.7)	0.2 (0.2)	1.1 (0.9)
Eichelborn	4.0 (8.1)	0.7 (1.9)	4.7 (10.0)	0.1 (0)	0.1 (0.1)	0.2 (0.1)
Groß Kreutz	1.1 (0.8)	1.6 (1.3)	2.7 (2.1)	0.7 (0.5)	1.3 (1.0)	2.0 (1.5)
Heidesee	6.2 (5.6)	7.1 (6.4)	13.3 (12.0)	3.8 (3.0)	21.6 (17.3)	25.4 (20.3)
Lychen	29.3 (19.2)	10.5 (7.6)	39.8 (26.8)	1.6 (0.9)	1.1 (0.7)	2.7 (1.6)
Müncheberg	2.1 (6.8)	0.5 (1.2)	2.6 (8.0)	0.8 (2.3)	0.1 (0.3)	0.9 (2.6)
Platkow	7.2 (8.5)	2.3 (3.6)	9.5 (12.1)	58 (61.1)	5.9 (8.0)	63.9 (69.1)
Quappendorf	1.1 (1.5)	2.7 (2.5)	3.8 (4.0)	0.2 (0.3)	0.9 (0.7)	1.1 (1.0)
Zülpich	4.2 (5.5)	2.5 (2.5)	6.7 (8.0)	0.4 (0.4)	0.5 (0.5)	0.9 (0.9)
Total	66.1 (65.4)	33.9 (34.6)	100 (100)	66.7 (69.4)	33.3 (30.6)	100 (100)

^a^Values in table are presented as the percentage of the total captured specimens at that location, with the relative percentage given in parentheses

A total of 794 blood-fed individuals from the Obsoletus Group and 958 blood-fed individuals from the Pulicaris Complex were captured, including at least one blood-fed female per trapping site (Table 4). Most blood-fed females (92.4%) were caught indoors, with the highest number recorded in Platkow, followed by the locations Eichelborn and Lychen (Table 4).

**Table 4:** Numbers of blood-fed females of the Obsoletus Group and Pulicaris Complex caught inside and outside the barns at the various study farms

Study farm	Inside barns			Outside barns		
	Obsoletus Group (*n*)	Pulicaris Complex (*n*)	Total (*n*)	Obsoletus Group (*n*)	Pulicaris Complex (*n*)	Total (*n*)
Beerfelde	19	2	21	0	0	0
Eggersdorf	40	12	52	0	0	0
Eichelborn	158	0	158	8	0	8
Groß Kreutz	1	1	2	1	0	1
Heidesee	53	47	100	14	57	71
Lychen	117	6	123	2	1	3
Müncheberg	4	0	4	1	0	1
Platkow	278	786	1064	2	39	41
Quappendorf	3	1	4	2	4	6
Zülpich	88	2	90	3	0	3
Total	761	857	1618	33	101	134

**Fig. 4:**
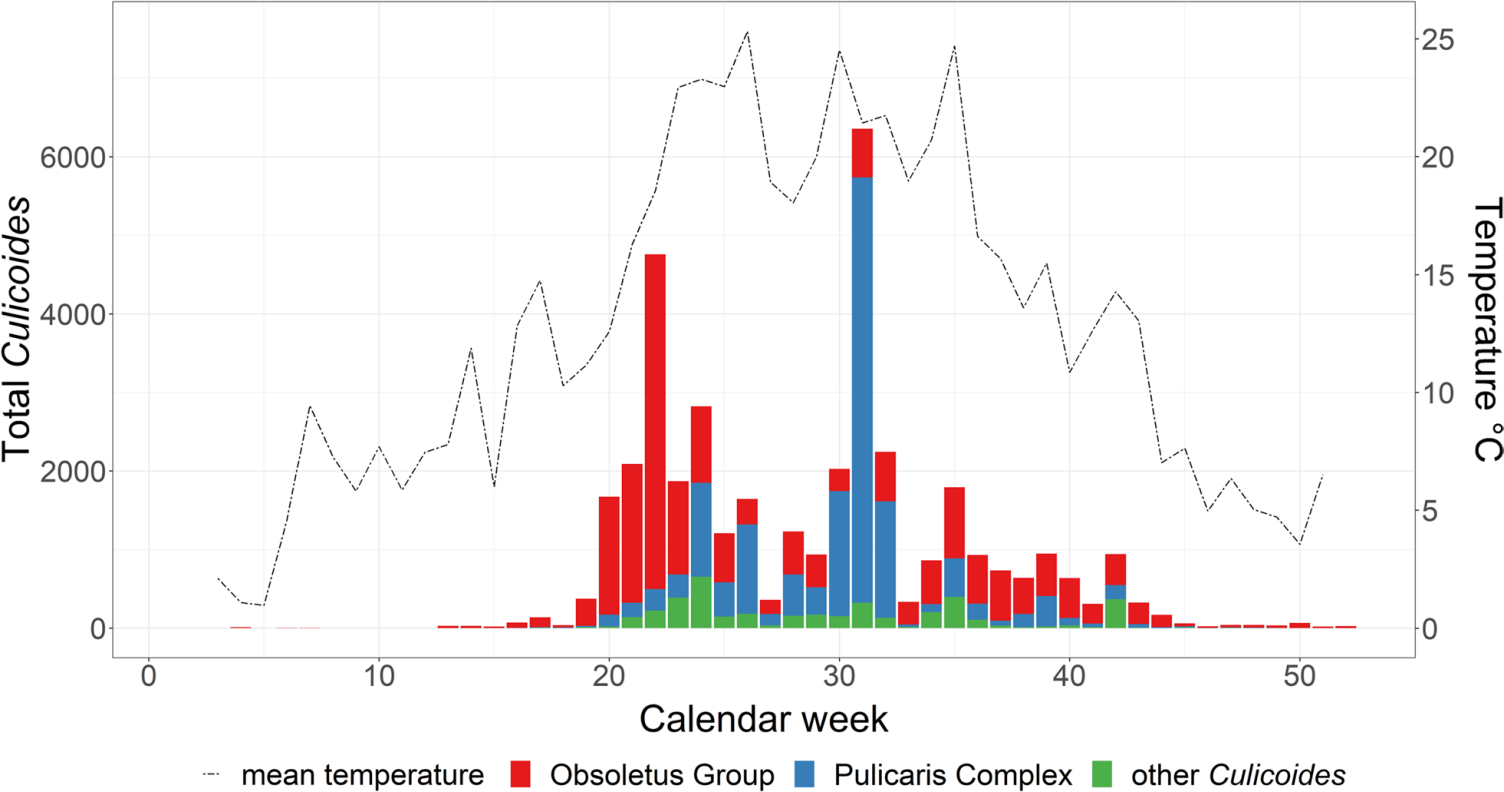
Total number of *Culicoides* specimens caught on the 10 farms in relation to the mean temperature measured at each trap

The numbers of total females, blood-fed females and males of the Obsoletus Group and Pulicaris Complex varied throughout the year, both inside and outside the barns, with most individuals caught between May and October (Table 5). With decreasing temperatures, the numbers of collected individuals decreased as well, with the highest collection numbers recorded between calendar week 20 in May and calendar week 35 in August (Fig. 4). The first and last seasonal Obsoletus Group catches were made at 10.7 °C (minimum 3.9 °C, maximum 23.2 °C) and approximately 75.6% relative humidity (minimum 61.0%, maximum 96.7%). For the Pulicaris Complex collection, the average threshold temperature was determined to be 12.8 °C (minimum 4.4 °C, maximum 24.9 °C) and the average relative humidity to be approximately 78.3% (minimum 53.0%, maximum 99.8%). Weekly temperature and relative humidity had no statistically significant effects on the total number of *Culicoides* collected (*t* = 1.80367 and *t* = 1.65711, respectively; both *P* > 0.05). However, the relative abundance of the Obsoletus Group was significantly correlated with temperature (*t* = 3.40309, *P* < 0.001), but not with relative humidity (*t* = 1.93182, *P* = 0.5338227). By contrast, the relative abundance of the Pulicaris Complex was significantly correlated with both temperature and relative humidity (*t* = 4.10764 and 3.47027, respectively; both *P* < 0.0001) (Table 6).

**Table 5:** Males/females of the Obsoletus Group and Pulicaris Complex per month, inside and outside the barns (all study farms)

Month	Inside barns^a^		Outside barns^a^		Total no. blood-fed females
	Obsoletus Group	Pulicaris Complex	Obsoletus Group	Pulicaris Complex	
January	0/7 (1)	0/0 (0)	0/7 (0)	0/0 (0)	1
February	1/4 (0)	0/0 (0)	0/4 (0)	0/0 (0)	0
March	0/25 (1)	0/2 (0)	1/6 (0)	0/0 (0)	1
April	16/127 (1)	0/4 (0)	26/73 (0)	0/8 (0)	1
May	12/6167 (157)	0/483 (17)	7/1716 (0)	3/147 (0)	174
June	93/1260 (96)	5/1556 (175)	53/1706 (1)	10/1496 (26)	298
July	320/714 (54)	16/1961 (321)	11/382 (1)	12/619 (5)	381
August	320/1559 (216)	24/5519 (301)	27/1094 (9)	36/1941 (55)	581
September	168/1253 (122)	2/256 (28)	45/792 (22)	13/552 (14)	186
October	44/892 (108)	2/216 (13)	10/637 (0)	13/144 (0)	121
November	1/56 (5)	0/11 (2)	1/67 (0)	4/13 (1)	8
December	2/77 (0)	0/4 (0)	0/64 (0)	0/0 (0)	0

^a^Values in table are presented as the numbers of male/female *Culicoides* biting midges, with the total number of blood-fed females in the respective months given in parentheses

**Table 6:** Coefficients and statistically significant output of the abiotic predictor variables ‘temperature’ and ‘relative humidity’ as calculated by the Poisson-generalised linear model

Abiotic predictor variables^a^	Estimate	Standard error	*t*-value	*P*
*Response variable a*				
Temperature	0.185839	0.103034	1.80367	0.071282
Relative humidity	0.078589	0.047425	1.65711	0.097498
*Response variable b*				
Temperature	0.094756	0.027844	3.40309	0.00066627***
Relative humidity	0.018293	0.009469	1.93182	0.05338227
*Response variable c*				
Temperature	0.506757	0.123369	4.10764	3.9972e−05***
Relative humidity	0.218053	0.062835	3.47027	5.1994e−04***

***Statistically significant

^a^Response variable ‘a’ is the sum of all *Culicoides* from all traps; response variable ‘b’ is the sum of Obsoletus Group specimens from all traps; response variable ‘c’ is the sum of Pulicaris Complex specimens from all traps

The presence of livestock had a significant impact on the relative abundance of the Obsoletus Group (*t* = 44.368, *P* < 0.0001) and ‘other *Culicoides*’ (*t* = − 36.004, *P* =  < 0.0001), but did not significantly affect the relative abundance of the Pulicaris Complex (*t* = 1.229, *P* = 0.219) (Table 7).

**Table 7:** Coefficients and statistically significant output of the predictor variable ‘presence of livestock’ as calculated by the Poisson-generalised linear model. The response variables are the sum of collected biting midges inside the barns of all study farms

	Estimate	Standard error	*t*-value	*P*
Inside	0.66578	0.01501	44.368	< 2e−16***
Pulicaris Complex	− 0.27884	0.01868	− 14.928	< 2e−16***
Other *Culicoides*	− 0.92384	0.02314	− 39.926	< 2e−16***
Inside × Pulicaris Complex	0.02815	0.02291	1.229	0.219
Inside × other *Culicoides*	− 1.31910	0.03660	− 36.044	< 2e−16***

***Statistically significant

No significant differences of the relative abundance of the Obsoletus Group, the Pulicaris Complex and ‘other *Culicoides*’ were found between barns with deep litter and barns with manure scraper or slatted floor (Additional file 2: Table S2).

## Discussion

By analysing systematic collection data, this study examines the seasonal activity of culicoid Obsoletus Group and Pulicaris Complex biting midges on 10 farms, both inside and outside barns in Germany.

Although the number of *Culicoides* individuals caught inside and outside barns varies from study to study, most authors report higher collection numbers inside than outside [26, 28, 33]. The same result was registered in the present study (63.1% of all individuals caught inside), with an inside:outside ratio of 1.7:1, a ratio that approximately corresponds to that obtained by Kameke et al. [31] (1.6:1).

In the present study, 23 different* Culicoides* species/species groups were caught outside the barns—almost twice as many as inside (13 species/species groups). This result is in line with findings reported by Baldet et al. [26], Romón et al. [33] and Sarvašová et al. [39], who also recorded higher species richness outside than inside barns in northern France, northern Spain and eastern Slovakia, respectively. The number of species mentioned in these studies varied, with the numbers identified in this study being somewhere in between. However, in the present study, the number of* Culicoides* species might actually well be higher than determined, as not all ‘other *Culicoides*’ could be morphologically identified to the species level. In fact, in addition to the Obsoletus Group and the Pulicaris Complex, ‘other *Culicoides*’ might also include species contributing to virus transmission. For example, the collections contained *Culicoides nubeculosus* (captured indoors and outdoors), which has previously been shown to be a competent BTV-vector [51, 52]. Other studies have discussed ‘other *Culicoides*’ species as potential vectors [53–55], but evidence is still missing.

The authors of various studies have raised the question of whether light trap catches underrepresent the abundance of some species of ‘other *Culicoides*’ [55–57]. If this were the case, vector species of this group might play a more important role in the occurrence and spread of *Culicoides*-borne diseases than commonly thought. Therefore, more efforts should be invested in the elucidation of the ecology of such ‘other *Culicoides*’.

Despite the high species diversity recorded in the present study, species of the Obsoletus Group and the Pulicaris Complex, particularly *C. obsoletus*/*C. scoticus*, *C. pulicaris* and *C. punctatus*, were the predominant species indoors and outdoors in the present study. Thus, more than half of the collected individuals could be assigned to the Obsoletus Group, while almost 40% were Pulicaris Complex specimens. Studies from central and northern Europe confirm the general high relative abundance of Obsoletus Group species [14, 28, 31, 32, 58], accounting for up to > 90% of the total catches [29]. The share in captured Obsoletus Group individuals in the present study, however, is lower than that recorded that in other investigations [14, 28, 29, 31, 32], probably due to an unexpectedly large number of Pulicaris Complex individuals being caught during several weeks at two of the 10 study farms (Heidesee and Platkow).

Previous studies have shown that the Pulicaris Complex was predominant at specific locations [59] or at certain times [28, 29, 39]. Mehlhorn et al. [29] suggest that the large number of Pulicaris Complex individuals during specific periods coincides with the simultaneous hatching of many individuals of this complex. This hypothesis might be supported by a study in Slovakia, during which extraordinarily high numbers of *C. punctatus*, a species of the Pulicaris Complex, were caught on a single trapping day [39], both indoors and outdoors. Based on the results of this study, a possible explanation for the locally and temporally high occurrence of Pulicaris Complex specimens appears to be the strong dependence on both temperature and relative humidity, while the activity of the Obsoletus Group was only correlated with temperature, as also reported in other studies [32, 39, 60]. To determine the true reasons for the restricted local and temporal peaks in the emergence of individuals of the Pulicaris Complex, further data are needed. Factors not investigated in the present study, such as land cover, soil type, soil moisture or vegetation, may be responsible.

It has been described that species of the Obsoletus Group tend to be endophilic while the Pulicaris Complex species *C. pulicaris* and *C. punctatus* rather appear to be exophilic [26, 40, 61]. Our results only partially support this notion, as considerable numbers of Obsoletus Group individuals were caught outdoors and many Pulicaris Complex individuals were caught indoors. However, these contradictory findings can possibly be attributed to the open construction of the barns which facilitated the easy entry and exit of biting midges. Meiswinkel et al. [40] also stresses that the building used in their study did not prevent *Culicoides* from getting inside. In addition, it has been suggested that a certain proportion of the *C. punctatus* population may adopt endophilic behaviour during their abundance peak [39].

In the present study, most of the indoor-collected Pulicaris Complex individuals were observed on two specific farms (Platkow and Heidesee). On the other study farms, relatively few individuals of the Pulicaris Complex were caught without a tendency for being predominantly collected outside or inside. Based on the literature [31, 56, 62], the types of livestock and animal husbandry practice might have affected the collection numbers of individuals of this complex, although these two parameters also differed between the locations Heidesee and Platkow (Heidesee: horse farm, 10 animals; Platkow: cattle farm, 820 animals). Moreover, the observation that such a high number of Pulicaris Complex specimens were trapped on individual days was not made on any other study farm that kept cattle or horses. Therefore, on the farms included in this study, factors other than the held animal species are likely to influence the behaviour of the Pulicaris Complex species. Different types of barn construction or landscape around the farms may affect the abundance and activity of *Culicoides* in general [31, 63]. However, these factors are not adequate to explain the high relative abundance or the behaviour of certain species in certain periods of the season.

The activity of *Culicoides* species varies during the year. For example, Clausen et al. [28] found different activity levels at different times of the year on German farms and, not surprisingly, observed reduced activity of the Obsoletus Group and the Pulicaris Complex during the winter. Mehlhorn et al. [29] also noted that, despite reduced activity, *C. obsoletus* specimens were caught during the winter. In this context, a possible vector-free period was brought into question [28]. In the present study, the Obsoletus Group was active both indoors and outdoors during all months of the year. Individuals of the Pulicaris Complex were active from March onwards but were also caught during the cold months until early December. Thus, our data support the existence of a vector-low, rather than a vector-free, period.

Biting midges are commonly collected by UV-light traps [29, 32, 35]. Using this approach, females are overrepresented over males [26, 32, 64], but at the same time are more important from a vector ecological point-of-view. As in other studies [26, 39, 40], in the present study the blood-fed females of the Obsoletus Group were mainly trapped indoors. While most of these females were caught during the summer months, Sarvašová et al. [39] reported a decrease in the number of blood-fed females in the barn to a negligible level at that time of the year. These authors assumed this decrease to be caused by the livestock being kept outdoors and the endophagic behaviour of the Obsoletus Complex individuals. The observation that the number of blood-fed Pulicaris Complex individuals caught indoors exceeded that of the Obsoletus Group in Platkow again contradicts the described exophagic behaviour of this complex [26]. On almost all farms included in our study, livestock was kept indoors even during summer nights, probably resulting in more blood-fed biting midge females caught indoors. In general, barns offer sheltered habitats for *Culicoides* species where they are not directly exposed to wind, precipitation and adverse temperatures [39]. These environmental parameters may decrease *Culicoides* activity outdoors [63], while individuals staying indoors remain active and are prone to being trapped [39].

The first catches of the Obsoletus Group and of the Pulicaris Complex during the study period were made at the end of January and at the end of March, respectively. The delayed activity of Pulicaris Complex individuals might be attributed to a higher threshold temperature, which was calculated to be 12.8 °C in the present study. By contrast, Kameke et al. [31] measured a threshold temperature of 10.9 °C for the Pulicaris Complex species *C. punctatus and C. pulicaris* [31]. Due to a lack of data, those authors could not calculate a threshold temperature for the Obsoletus Group. However, based on the observation that the Obsoletus Group was active sooner in the season, they concluded that the threshold temperature had to be higher for the Pulicaris Complex than for the Obsoletus Group [31]; it would appear that our results confirm this assumption.

Silbermayr et al. [65] correlated the occurrence of the genus *Culicoides* with weather data and identified a significant influence of temperature and relative humidity in Austria, which we could confirm only for the Pulicaris Complex, but neither for the genus *Culicoides* in general nor for the Obsoletus Group in particular. However, in many other studies, all *Culicoides* species were considered as a whole [32, 39, 62]. Further studies that have demonstrated a correlation between the abundance of *Culicoides* species and temperature and relative humidity focus on species that do not occur in Germany, such as *C. imicola* [66, 67], or which were conducted in other climatic zones [65, 67] or under constant laboratory conditions [66, 68].

The present study was also able to prove statistically that the ‘presence of livestock’ influenced the number of caught individuals of the Obsoletus Group. As opposed to that, ‘presence of livestock’ does not appear to affect the activity of the Pulicaris Complex. This result is in agreement with the stenoecious behaviour of the Pulicaris Complex [69].

Finally, the impact of deep litter, which was removed once or twice a year, and a manure scraper or slatted floor cleaning system on the presence of *Culicoides* biting midges was examined. Between these two husbandry approaches, there were no significant differences in the catch numbers of either the Obsoletus Group or the Pulicaris Complex. At least for the Obsoletus Group, this result was surprising as some members of this group have been described as preferring dung and manure for larval development [37, 70, 71]. However, on farms examined in the present study that were equipped with manure scrapers or slatted floors, the manure was not completely removed from the barns or their vicinity. According to the farmers, the scraped-up manure was stored in pits under or near the barn or fell into an open pit under the slatted barn floor. Thus, the manure was still available as a breeding habitat until the pits were cleaned out. However, as the results from only nine traps and farms were used for this analysis, further investigations are necessary to make a well-founded statement on whether the husbandry system influences the occurrence of *Culicoides*.

The considerable duration of seasonal activity of putative *Culicoides* vector species inside and outside barns demonstrates the importance of spatially and temporally more differentiated analyses of the risk of virus transmission by *Culicoides* biting midges. It also highlights details on which further research is needed. In addition, the presence of host-seeking *Culicoides* females on all farms indicates that extensive virus transmission and disease risk can be inferred, provided the virus circulates in the study regions.

## Conclusion

Most specimens collected in the present study belonged to the Obsoletus Group and Pulicaris Complex of the ceratopogonid genus *Culicoides*, which are believed to contain the major vectors of SBV and BTV. These two species groups/complexes were caught on all livestock farms investigated in the present study, highlighting the risk of virus transmission if the virus becomes introduced into Germany. Compared to the Pulicaris Complex, individuals of the Obsoletus Group have a lower threshold temperature for activity and were caught from January to December. Individuals of both the Obsoletus Group and the Pulicaris Complex were caught inside and outside barns, suggesting that virus transmission in and around the barns is possible even in winter. Due to highest collection numbers of *Culicoides* from late May to August, the transmission risk appears to be the highest during this time period. Winter activity, however, was also observed and should be studied more closely. In addition, further investigations should be considered to explain the sudden high occurrence of the Pulicaris Complex at certain times and locations and to examine the ecology and epidemiological role of *Culicoides* species not belonging to the Obsoletus Group and the Pulicaris Complex.

### Supplementary Information

**Additional file 1: Table S1.** Numbers and percentages (in brackets) of *Culicoides* and other biting midges captured with UV-light traps inside and outside the barns on all study farms.

**Additional file 2: Table S2.** Non-significant results of the two sample t-tests comparing the numbers of *Culicoides* caught in the barns with deep litter, manure scraper or slatted floor.

## Data Availability

All data supporting the conclusions of this article are included within the article and in its additional files.

## Abbreviations

BTV: Bluetongue virus
ELISA: Enzyme-linked immunosorbent assay
GLM: Generalised linear model
LU: Livestock unit
SBV: Schmallenberg virus
UV: Ultraviolet
