# Supplementary material for: Occurrence of putative Culicoides biting midge vectors (Diptera: Ceratopogonidae) inside and outside barns in Germany and factors influencing their activity
Source: Parasit Vectors. 2023 Aug 31;16:307. doi: 10.1186/s13071-023-05920-z (PMC10472570; doi:10.1186/s13071-023-05920-z)
Supplement: Supplementary file 1 — Additional file 1: Table S1. Numbers and percentages (in brackets) of Culicoides and other biting midges captured with UV-light traps inside and outside the barns on all study farms. [file 13071_2023_5920_MOESM1_ESM.docx]

**Additional file 1: Table S1**

| Trap position | Inside | | Outside | | Total | |
| --- | --- | --- | --- | --- | --- | --- |
|  | Male | Female | Male | Female | Male | Female |
| Obsoletus Group | 977 (7.4%) | 12,141 (92.6%) | 181 (2.7%) | 6,548 (97.3%) | 1,158 (5.8%) | 18,689 (94.2%) |
| Pulicaris Complex | 49 (0.5%) | 10,012 (99.5%) | 91 (1.8%) | 4,920 (98.2%) | 140 (0.9%) | 14,932 (99.1%) |
| other *Culicoides* | 60 (4.4%) | 1,314 (95.6%) | 172 (6.6%) | 2,421 (93.4%) | 232 (5.8%) | 3,735 (94.2%) |
| total *Culicoides* | 24,553 | | 14,333 | | 38,886 | |
