# Supplementary material for: Occurrence of putative Culicoides biting midge vectors (Diptera: Ceratopogonidae) inside and outside barns in Germany and factors influencing their activity
Source: Parasit Vectors. 2023 Aug 31;16:307. doi: 10.1186/s13071-023-05920-z (PMC10472570; doi:10.1186/s13071-023-05920-z)
Supplement: Supplementary file 2 — Additional file 2: Table S2. Non-significant results of the two sample t-tests comparing the numbers of Culicoides caught in the barns with deep litter, manure scraper or slatted floor. [file 13071_2023_5920_MOESM2_ESM.docx]

**Additional file 2: Table S2**

|  | M Deep litter | M Scraper | t (df) | *P* |
| --- | --- | --- | --- | --- |
| Obsoletus Group | 6.51611667 | 0.0839364 | 1.8171 (7) | 0.112 |
| Pulicaris Complex | 0.7677600 | 0.1670714 | 0.99393 (7) | 0.3534 |
| Other *Culicoides* | 0.41111167 | 0.06090041 | 0.9487 (7) | 0.3744 |
